# Muscle Oxygenation Measured with Near-Infrared Spectroscopy Following Different Intermittent Training Protocols in a World-Class Kayaker—A Case Study

**DOI:** 10.3390/s22218238

**Published:** 2022-10-27

**Authors:** Rūtenis Paulauskas, Ričardas Nekriošius, Rūta Dadelienė, Ana Sousa, Bruno Figueira

**Affiliations:** 1 Educational Research Institute, Education Academy, Vytautas Magnus University, 44244 Kaunas, Lithuania; 2 Department of Applied Biology and Rehabilitation, Lithuanian Sport University, 44221 Kaunas, Lithuania; 3 Institute of Health Science, Department of Rehabilitation, Physical and Sports Medicine, Vilnius University, 01513 Vilnius, Lithuania; 4 Research Center for Sports, Exercise and Human Development, University of Trás-os-Montes e Alto Douro, 5000-801 Vila Real, Portugal; 5 Research Center for Sports, Exercise and Human Development, University of Maia, ISMAI, 4475-690 Maia, Portugal

**Keywords:** oxygen saturation, total hemoglobin, intensity, exercise bouts, recovery

## Abstract

Training elite kayakers at a distance of 1000 m is associated with aerobic and anaerobic metabolism, while intermittent training, in a variety of forms, is one of the effective ways to improve cardiorespiratory and metabolic function. Thus, this study aimed to investigate muscle oxygenation responses during repetition training (RT), interval training (IT), and sprint interval training (SIT). Near-infrared spectroscopy (NIRS) monitors were placed on the latissimus dorsi (LD), pectoralis major (PM), and vastus lateralis (VL) of a world-class kayaker during their preparatory period. The intensity of work, relief, and recovery intervals were the independent variables that were manipulated using three different training protocols. The inferential analysis between intermittent training protocols showed significant differences for all variables except total the hemoglobin (tHb) index in LD during bout 2 (F = 2.83, *p* = 0.1, ηp2 = 0.205); bout 3 (F = 2.7, *p* = 0.125, ηp2 = 0.193); bout 4 (F = 1.8, *p* = 0.202, ηp2 = 0.141); and bout 6 (F = 1.1, *p* = 0.327, ηp2 = 0.092). During the rest bouts, all training protocols showed significant differences for all variables except muscle oxygen saturation (SmO_2_) in the VL during bout 5 (F = 4.4, *p* = 0.053, ηp2 = 0.286) and tHb in VL during bout 1 (F = 2.28, *p* = 0.132, ηp2 = 0.172); bout 2 (F = 0.564, *p* = 0.561, ηp2 = 0.049); bout 3 (F = 1.752, *p* = 0.205, ηp2 = 0.137); bout 4 (F = 1.216, *p* = 0.301, ηp2 = 0.1); and bout 6 (F = 4.146, *p* = 0.053, ηp2 = 0.274). The comparison between IT protocols RT and SIT presented similar results. All variables presented higher values during SIT, except HR results. Finally, the comparison between IT and SIT showed significant differences in several variables, and a clear trend was identified. The results of this study suggest that the application of different intermittent exercise protocols promotes distinct and significant changes in the peripheral effect of muscle oxygenation in response to training stimuli and may be internal predictors of hemodynamic and metabolic changes.

## 1. Introduction

To achieve high-standard-specific goals, athletes are constantly looking for ways to optimize skeletal muscle function and its monitoring process through different types of physical training methodologies and evaluation procedures. Based on oxygen-dependent characteristics, NIRS is one of the non-invasive methods that can provide information about the changes in the oxygen saturation of muscle tissue during various sports exercises [1]. For that reason, the popularity of the NIRS method in sports research and real-world scenarios has been growing in recent years [2]. Wearable and wireless devices are fixed in the muscle, and shining near-infrared light travels along the tissue [3,4]. Light passes through muscles, especially in the near-infrared region of the spectrum, and differences in the reflection index within cells promote scattering [5]. Thus, NIRS technology can supply information on SmO_2_ and the tHb index in real time [4,5]. Considering that more than 90% of the total blood volume in the muscle is supplied by capillaries and that under regular conditions, all muscle tissues receive fully oxygenated arterial blood, NIRS technology can detect changes in capillary hemoglobin and intracellular myoglobin oxygen levels [6]. The validity of NIRS technology was established through the strong correlations with venous O_2_ saturation, not only during exercise but also during rest (r = 0.92) [7]. Additionally, NIRS measurements are highly reproducible during repeated incremental running (r = 0.87–0.88) and cycling (r = 0.94–0.99) tests to exhaustion [8]. Several studies have focused on the application of NIRS in assessing the effectiveness of sports training programs [9], and the effects of endurance, interval, and hypoxic training on muscle oxygenation in trained and untrained subjects [10]. Ferrari, Muthalib [4], and Hamaoka and McCully [5] reviewed NIRS methodologies and found that most studies focused on endurance or strength training and related to muscle oxidative capacity, oxygen delivery, and oxygen consumption. Only four studies on oxygenation profiles have been completed [11,12] utilizing wearable NIRS devices on well-trained kayakers. Based on the current literature, NIRS appears to have the potential to contribute to human performance analysis. It presents an opportunity to evaluate muscle contribution to the athlete’s motion and the ability to sustain performance, which will directly influence fatigue during exercise [13].

Even though continuous endurance training and high-intensity interval training can promote maximal oxygen (VO_2_max) uptake adaptations in subjects with low training status, VO_2_max improvements are greater after interval training modes across the lifespan [14]. 

Currently, there is great interest in different intensity intermittent training programs [15] in which the manipulation of the exercise intensity as well as the duration and recovery regimen remain the variables that determine the total workload [16]. Based on these variables, the concepts of RT, IT, and SIT emerged in sports training practice and raise problematic issues related to muscle metabolism that require scientific analysis. RT induces numerous physiological adaptations that facilitate improved exercise capacity, i.e., the ability to sustain a given workload for an actual period of time or achieve a higher average power output over a fixed distance or time [17,18]. The principle of IT was first described by Reindell and Roskman [19] and is a method of training that alternates between exercise periods and recovery and with specific adaptive effects on the athlete’s heart. In moderate-intensity interval training, when alternating 30–90 s exercise that reaches the anaerobic threshold, the systolic volume of the heart increases during recovery intervals, causing myocardial metabolic load [20]. Interval training can be performed effectively with numerous combinations of work duration and intensity, rest duration, and intensity, and their effects on aerobic exercise training are still being studied [21]. SIT is characterized by efforts performed at intensities equal to the pace that would elicit VO_2_max, including the maximal efforts of the athlete [22]. This training increases the maximal activities of mitochondrial enzymes [23], reduces glycogen utilization and lactate accumulation during matched-work exercise [24,25], and improves performance during those tasks that primarily rely on aerobic metabolism [25]. 

Flat water race 1000 m kayaking is a highly demanding sport that requires both aerobic and anaerobic efforts, which reinforces the importance of selecting adequate methods of endurance training. The aerobic contribution, expressed as a fraction of VO_2_max, was shown to be 85% for 1000 m kayaking and lasting approximately 3 min 45 s [26]. Kayakers of the Olympic standard were reported to reach a peak oxygen uptake from 4.27 to 4.67 L·min^−1^ during an on-water 1000 m race [27,28]. One of the recent studies [29] characterized the changes in oxygenation derived from NIRS in three active muscles during a VO_2_max test and on-water time trials (200, 500, or 1000 m) in male and female U23 and senior athletes and found the relation between muscle oxygenation, VO_2_max, maximal cardiac output, and performance. The analysis of stroke force data and electromyography (EMG) revealed that the most active muscles during kayak stroke are the anterior deltoid (AD), triceps brachii (TB), LD, and VL [30]. Thus far, there is only one study in junior male athletes that displayed a moderate correlation between the maximal O_2_ extraction in the LD during an incremental test on a kayak ergometer and both 200 and 1000 m performances [15]. It is understood that some muscle oxygenation studies are limited by technical conditions that cause inconvenience in attaching sensors and performing paddling movements. However, there is a need to determine the contribution of other muscles, such as the PM, that have not been previously studied in any kayak training protocol. Depending on the demands of the training, several approaches exist to control and individualize intermittent exercise intensities [31,32]. Individual incremental test parameters are much more objective, practical, and likely the more accurate and effective criteria for achieving the desired performance results [33,34,35]. Paquette and Bieuzen [36] studied thirteen canoe kayak athletes of different genders and different levels to determine their muscle oxygenation and cardiac output responses to various HIIT sessions with the intensity ranging from 110% peak power output to all-out, suggesting that the muscle demand for O_2_ is high, especially with the increase in the number and targeting intensity. However, it is unknown whether moderate-intensity repetition work can cause muscle oxygenation and cardiac output in world-class kayakers, compared with moderate interval and sprint interval training. Thus, the purpose of this study is to assess muscle oxygen responses during the RT, IT, and SIT in a world-class kayaker and to determine oxygenation parameters in the VL, PM, and LD muscles in each training workout. We hypothesized that the SIT training protocol activates the muscle’s O_2_ dynamics and oxidative energy metabolism more than the IT and RT, and this response would be detected using NIRS.

## 2. Materials and Methods

### 2.1. Subject

A male world-class kayaker (World Championship silver and bronze medal winner and European Championship bronze medal winner in 1000 m kayak flat water race event), during the preparatory period, participated in this study. At the start of the data collection, the participant’s age was 32 years, with a height of 184.5 cm, body mass of 89 kg, and training volume of 18 h·week^−^^1^. Physical characteristics are presented in Table 1. 

### 2.2. Design

During the study, the athlete was encouraged to undertake their normal training but not to train on the day before each test. The athlete was acquainted with the experimental procedures prior to testing and gave written informed consent to participate in the study. All the experimental procedures involved in this study were approved by the Bioethics Research Committee of Vilnius Region (#158200-18/11-1040-573). This study adhered to ethical principles under the Declaration of Helsinki.

The participant performed three randomized separated training sessions, RT, IT, and SIT, upon a Dansprint PRO, KE001 ergo, Denmark kayak ergometer at air brake resistance level 7. The ergometer was calibrated before each test according to the manufacturer’s recommendations, and the tension in the ergometer’s ropes was verified regularly [37]. All sessions were performed under similar environment conditions (relative humidity 60%) and circumstances (from 11.00 to 12.30 h).

The study protocol practice sessions started with a 15 min standard warm-up comprising rowing exercises and 5 min of recovery (Table 2).

For all tests, the athlete started in the kayak ergometer, waited 3 min for the starting signal, and established the baseline responses for NIRS and HR monitor. The participant was asked to perform the same volume of 6 bouts of activity interspersed with 6 min of passive recovery (Table 2). To program the RT, IT, and SIT intensities, the subject’s individual power output characteristics were used at the critical intensity limit (CIL) and at the second ventilatory threshold (VT2) associated with physiological markers such as VO_2_max, VO_2_, and HR (Table 1) [38]. The participant received stroke power output and heart rate (HR) feedback during the test and was asked to maintain the right intensity (W) in each stage of the exercises. Blood lactate (Bla) concentration (mmol·L^−1^), as a proxy for metabolic anaerobic demand, was determined 3 min after the end of each intermittent training.

### 2.3. Protocols

#### 2.3.1. RT

The RT protocol comprised 6 bouts of 6 min of ergometer paddling at 200 watts intensity interspersed with 6 min of passive recovery. The participant was instructed to assume the ready position, and after the starting signal, the activity lasted for 72 min. 

#### 2.3.2. IT

The IT protocol comprised 6 bouts of 6 min that consisted of interspersing periods of 1-min ergometer paddling at the intensity of 200 watts and 1 min relief at 40 watts paddling intensity. The 6 min of activity were interspersed with 6 min of passive recovery. The participant was instructed to assume the ready position, and after the starting signal, the activity lasted for 72 min.

#### 2.3.3. SIT

The SIT protocol comprised 6 bouts of 6 min that consisted of interspersing periods of 10 s of ergometer paddling at 300 watts intensity with 30 s of relief paddling at 40 watts intensity. The 6 min of activity were interspersed with 6 min of passive recovery. The participant was instructed to assume the ready position, and after the starting signal, the activity lasted for 72 min.

### 2.4. Variables

#### 2.4.1. NIRS Values

**Figure 1:**
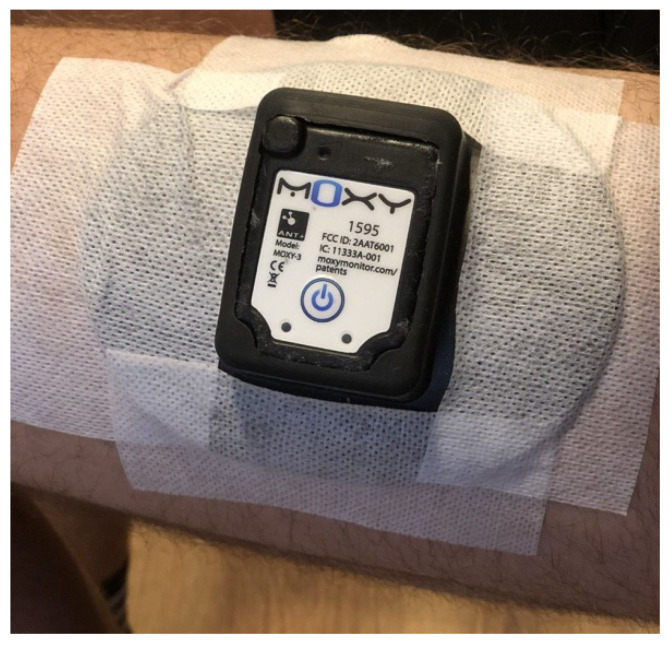
Moxy monitor placement.

The oxygenation level of exercising muscles (oxygenated hemoglobin), SmO_2_ (%), and deoxygenated total hemoglobin, tHb (arbitrary units AU), were assessed with a NIRS device (Moxy Oxygen Monitor-USA, Hutchinson, MN, USA) (Figure 1).

Three NIRS monitors were placed and affixed using double-sided adhesive tape over the left (dominant) VL, PM, and LD muscles: for the VL, on the distal part of the VL muscle belly (10 cm above the proximal border of the patella); for the PM, on the center of the muscle belly along in the principal direction of the muscle fibers of the sternocostal head, and for the LD, on the midpoint between the inferior border of the scapula and the posterior axillary fold. The skinfold thickness at each site was measured using a skinfold caliper (Harpenden, C-136) to ensure that the skinfold thickness was less than half the distance between the emitter and the detector (25 mm). The raw muscle O_2_ saturation (SmO_2_) and total hemoglobin concentration (tHb) signals were captured at 10 Hz, and the data were smoothed using a 10th order low pass-zero phase Butterworth filter (cut-off frequency 0.1 Hz) provided by the recording Artinis Software (Oxysof, Artinis Medical System, Elst, The Netherlands) [39]. Black elastic bandages were used to shield the probes from ambient light and minimize movement during exercise. The values of muscles oxygenation at the baseline (averaging 30 s before exercise), during exercise (sample size of each bout *n* = 180), and during exercise recovery periods (sample size of each bout *n* = 180) were recorded in the Moxy PC software (Fortiori Design LLC, Minneapolis, MN, USA), which allowed for the calculation of the average of the recorded values and the lowest point of the SmO_2_ in each training. The variation between recovery and exercise in SmO_2_ (Δ SmO_2_) was calculated by evaluating the difference between the minimum SmO_2_ and baseline SmO_2_, and the tHb (Δ tHb) variation was also assessed by calculating the variation between the maximum tHb and baseline tHb [40].

#### 2.4.2. Heart Rate Responses

HR responses were assessed with a telemetric HR monitor (Polar RS800 CX, Polar Electro Oy, Kempele, Finland). The HR (sample size of each bout *n* = 180) was measured during all the interval bouts, including during the rest. The HR signals were treated using a moderate filter, cleaning and replacing all irregular heartbreaks with interpolated, adjacent R–R interval values using the Polar Software (Pro Trainer 5, Polar Electro, Finland).

#### 2.4.3. Blood Lactate Concentration

Blood lactate (Bla) concentration (mmol·L^−1^) was calculated 3 min after the end of the protocols. The blood lactate samples were taken from the participant’s fingertip and immediately analyzed with a validated lactate analyzer (Lactate Pro; Arkray, Tokyo, Japan).

### 2.5. Statistical Analysis 

Descriptive analysis is presented in Table 3 and Table 4, and data are presented as means (M)  ±  standard deviations (SD). Before using the parametrical statistical procedures, the assumptions of normality and sphericity were verified. A one-way repeated-measure ANOVA was performed to identify the differences in muscle oxygen saturation and the total hemoglobin in the VL, PM, and LD muscles, and the heart rate between the interval training modes. Bonferroni’s corrections were used for the comparisons of more than two groups, and Cohen’s d was calculated as the effect-size measure. The alpha level for all statistical tests was set a priori at α = 0.05, and the calculations were carried out using the SPSS software V24.0 (IBM SPSS Statistics for Windows, Armonk, NY, USA: IBM Corp.). The thresholds for effect-size statistics were <0.2, trivial; <0.6, small; <1.20, moderate; <2.0, large; and >2.0, very large. These statistical computations were processed with a specific post-only crossover spreadsheet for each age group [41].

## 3. Results

**Figure 2:**
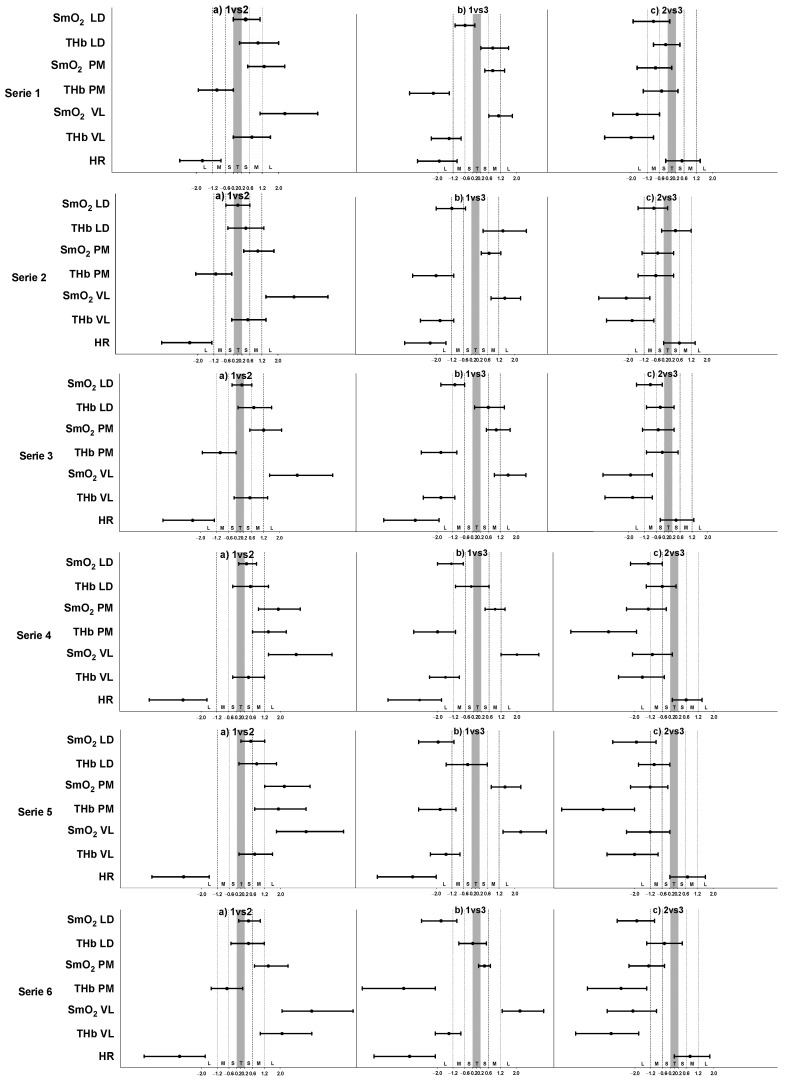
Standardized (Cohen) differences in the considered variables during exercise bouts. Error bars indicate uncertainty in the true mean changes with 95% confidence intervals. Abbreviations: 1—RT protocol 1; 3—IT protocol 2; SIT protocol 3; SmO_2_—muscle oxygen saturation; tHb—total hemoglobin; LD—latissimus dorsi; PM—pectoralis major; VL—vastus lateralis; HR—heart rate; T—trivial effect; S—small effect; M—moderate effect; L—large effect.

**Figure 3:**
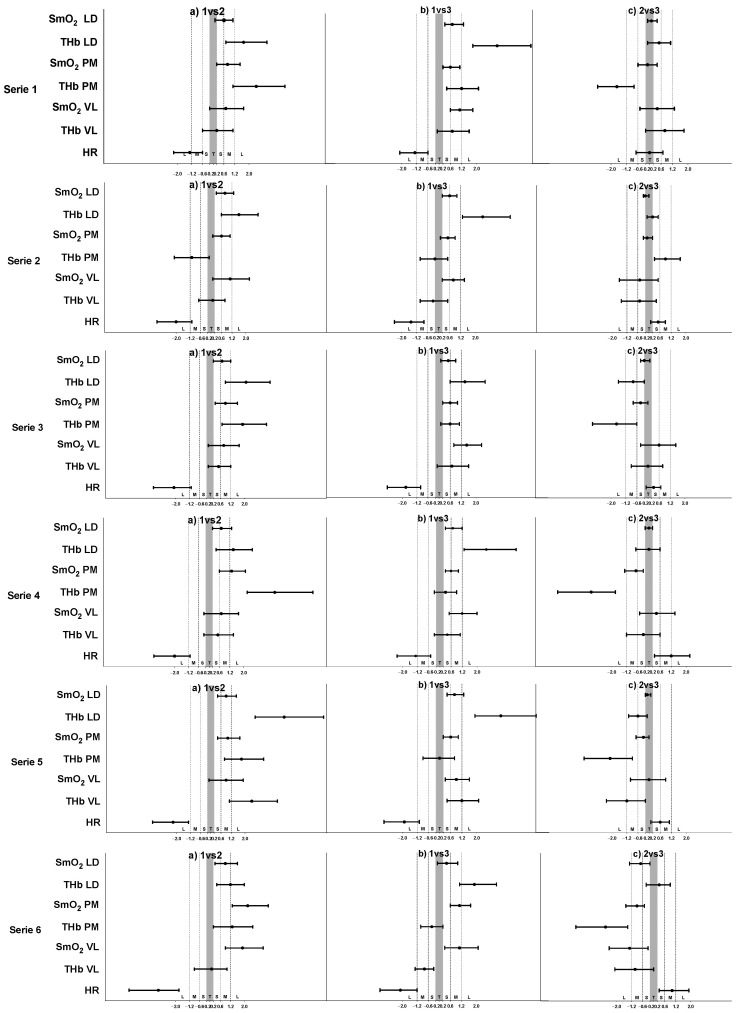
Standardized (Cohen) differences in the considered variables during rest bouts. Error bars indicate uncertainty in the true mean changes with 95% confidence intervals. Abbreviations: 1—RT protocol 1; 3—IT protocol 2; SIT protocol 3; SmO_2_—muscle oxygen saturation; tHb—total hemoglobin; LD—latissimus dorsi; PM—pectoralis major; VL-vastus lateralis; HR—heart rate; T—trivial effect; S—small effect; M—moderate effect; L—large effect.

The results of the inferential analysis between the intermittent training protocols during the exercise bouts and rest bouts are presented in Table 3 and Table 4, respectively. Complementarily, both figures show the standardized (Cohen) differences for the pairwise comparisons. During the exercise bouts, all the intermittent training protocols presented significant differences for all variables except tHb in the LD during bout 2 (F = 2.83, *p* = 0.1, ηp2 = 0.205); bout 3 (F = 2.7, *p* = 0.125, ηp2 = 0.193); bout 4 (F = 1.8, *p* = 0.202, ηp2 = 0.141); and bout 6 (F = 1.1, *p* = 0.327, ηp2 = 0.092). During the rest bouts, all the intermittent training protocols showed significant differences for all variables except SmO_2_ in the VL during bout 5 (F = 4.4, *p* = 0.053, ηp2 = 0.286) and the tHb in the VL during bout 1 (F = 2.28, *p* = 0.132, ηp2 = 0.172); bout 2 (F = 0.564, *p* = 0.561, ηp2 = 0.049); bout 3 (F = 1.752, *p* = 0.205, ηp2 = 0.137); bout 4 (F = 1.216, *p* = 0.301, ηp2 = 0.1); and bout 6 (F = 4.146, *p* = 0.053, ηp2 = 0.274). Complementarily, Figure 2 and Figure 3 show the standardized (Cohen) differences for the pairwise comparisons. The comparison between the protocols showed that the RT protocol presented higher deoxygenation levels than the IT protocol. On the other hand, the SIT protocol presented higher deoxygenation levels than the RT and IT protocols but only in the LD muscle (Figure 2).

However, the SIT and IT protocols presented higher mean O_2_ saturation levels during the passive recovery than the RT protocols.

## 4. Discussion

Our research aimed to assess muscle oxygenation responses during the RT, IT, and SIT in a world-class kayaker and to determine their parameters in the VL, PM, and LD muscles. The findings of this case study only partially confirm our hypothesis: (1) the RT was characterized by a greater mean deoxygenation rate than the IT protocol, and the SIT mean deoxygenation was greater than the RT and IT workouts only in the LD muscle; however, (2) the mean O_2_ saturation level during the passive rest period was higher in the SIT and IT protocols than in the RT; (3) oxygenation responses in the three active muscles suggest higher PM muscle recruitment than those of the LD and VL muscles as well as changes in the level of muscle contribution during the exercises of different intensities. This study shows the possibilities of using NIRS devices in the monitoring of elite kayak paddling performance and may provide complementary information to the HR and Bla concentration on a local muscle metabolism level. 

### 4.1. O_2_ Dynamics during Different Training

In this study, we identified oxygen muscle changes during the RT, IT, and SIT protocols. The RT induced greater mean oxygenation in the PM and VL and caused a greater HR response than other protocols we applied. In our design, the RT involved a constant intensity of around ~65% of the critical intensity limit (CIL) when performing a 200 W workload. The increase in mean HR during the RT may reflect an increase in the cardiac output associated with a central cardiocirculatory component of the training [42]. IT intensities (200 W) were similar to RT intensities; however, relief intervals reduced the mean oxygenation level of the IT, which had the lowest Δ SmO_2_ (%) and the highest Δ tHb during all exercise bouts when compared with the RT and SIT protocols (Table 5). 

**Figure 4:**
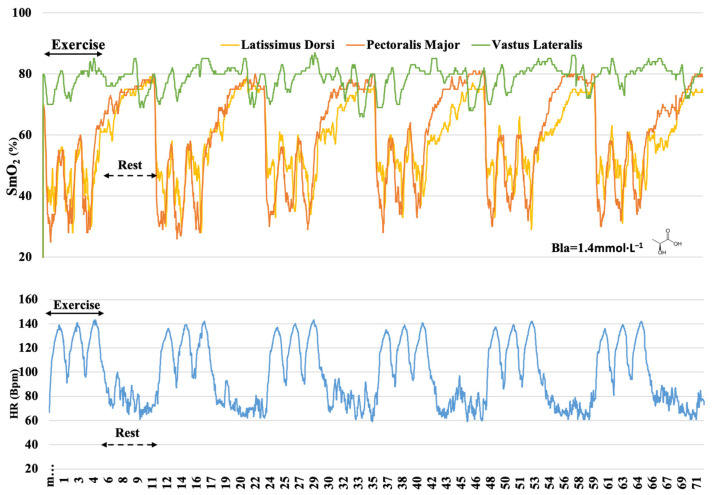
Muscle oxygen saturation (SmO_2_) kinetics in latissimus dorsi, pectoralis major, and vastus lateralis and heart rate (HR) response during IT protocol; blood lactate (Bla) concentration 3 min after the end of the protocol.

The RT was distinguished by the duration of continuous work, while the SIT featured the increased intensity of short intervals. The reviewers of this type of RT response categorize them as metabolic, eliciting large requirements from the O_2_ transport and utilization systems [43], and responses to protocols such as the SIT are considered metabolic but with a certain degree of neuromuscular strain [21]. Previous reports state that during moderate-intensity IT, the systolic volume of the heart increases during recovery intervals, causing myocardial metabolic load [21]. Therefore, hypothetically, peripheral metabolic changes were not expected in our study. Paquette and Bieuzen [40] considered that Δ SmO_2_ is a good performance predictor since SmO_2_ represents the balance between O_2_ delivery and extraction at the muscle level [4]. Thus, a decrease in SmO_2_ may originate from both reduced delivery and/or increased extraction. However, it is difficult to draw a conclusion indicating the importance of the exercise mode to elicit the cardiovascular component. The control or adjustment of the intensity of the training sessions related to HR may be limited due to the well-known HR delay at exercise onset [20], which showed a slower response than the SmO_2_ response during the IT protocol (Figure 4. As the oxygen demand in the working muscle is the driving force for oxygen delivery by the cardiovascular system [42], muscle deoxygenation responded even faster than the oxygen uptake to the onset of a time trial [44].

Judging by the NIRS indicators, in the LD, the peripheral effects on oxygen extraction during the SIT protocol were higher than during the RT. It has been previously established that increasing exercise intensity improves aerobic energy metabolism, which is primarily linked to increased skeletal muscle mitochondrial content and capillary density [45].

**Figure 5:**
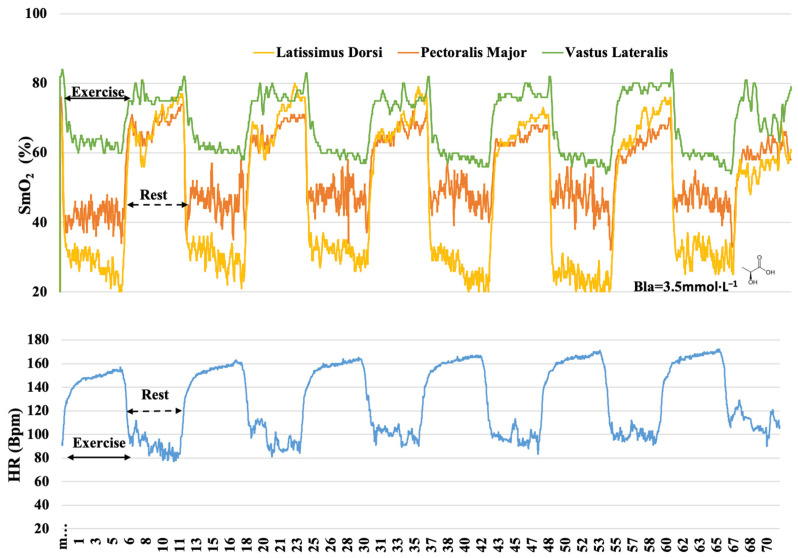
Muscle oxygen saturation (SmO_2_) kinetics in latissimus dorsi, pectoralis major, and vastus lateralis and heart rate (HR) response during RT protocol; blood lactate (Bla) concentration 3 min after the end of the protocol.

**Figure 6:**
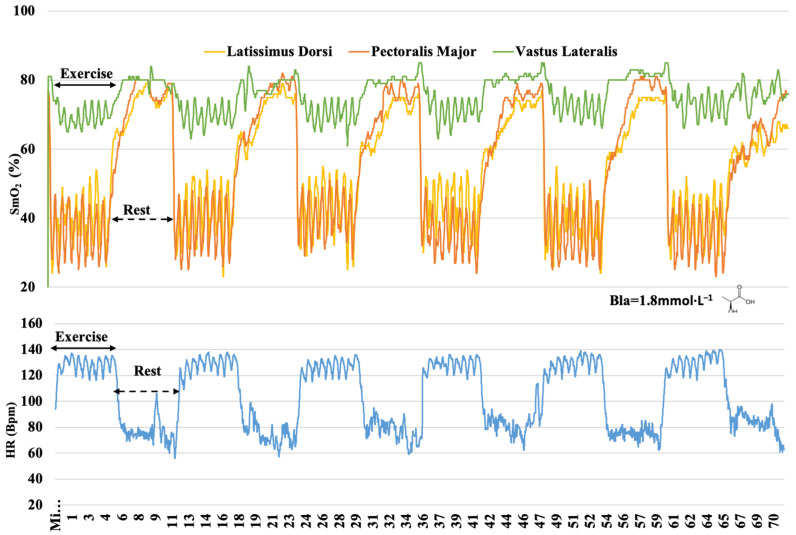
Muscle oxygen saturation (SmO_2_) kinetics in latissimus dorsi, pectoralis major, and vastus lateralis and heart rate (HR) response during SIT protocol; blood lactate (Bla) concentration 3 min after the end of the protocol.

Another feature of our study was to monitor the mean muscle oxygenation of each training session in 6 min duration rest bouts (Figure 4, Figure 5 and Figure 6). It was possible to observe after which training protocol O_2_ returned faster to the pre-exercise conditions since recovery is an important component to improve physical training adaptations [46]. In the RT, oxygenation during the rest bouts in the PM ranged from 54.4% to 65.9%, in the IT from 72% to 79.5%, and in the SIT from 63.5% to 78.6% of SmO_2_ (Table 3). Our findings suggest that the link between the O_2_ uptake recovery might be related to the exercise intensity and the nature of repeated sequences in the IT and SIT. The ability to resist fatigue (SmO_2_% decrement) and replenish the energy substrates (ATP and PCr) are oxygen-dependent processes [47]. In the present study, during the rest bouts, muscle oxygenation in different training protocols returned at a different pace to pre-exercise (~80%) levels (Figure 4, Figure 5 and Figure 6), indicating a possible recovery of muscle PCr [48]. The VL muscle was the least affected, presenting oxygenation at its highest level, and in the RT, the mean values ranged from 71.1% to 76.5%, while in the IT, it ranged from 77% to 81%, and in the SIT, from 76.3% to 80.8% of SmO_2_ (Table 4). Different levels of muscle recovery may be related to factors such as impairments in neural drive and motor unit activation or metabolite accumulation [49]. The oxygenation in the muscles quickly adjusted post-exercise, indicating that the use of NIRS technology showed high sensitivity and may lead to discussion and further investigations as to whether oximetry and HR monitoring are more sensitive methods, especially in the IT (Figure 4) and SIT (Figure 6). The three training protocols elicited different increases in blood lactate concentrations during the exercise, showing the contribution of the anaerobic glycolytic system, inferred by blood lactate accumulation, to be numerically greater in the RT (3.5 mmol·L^−1^) (Figure 5) than in the IT (1.4 mmol·L^−1^) (Figure 4) and SIT (1.8 mmol·L^−1^) (Figure 6). The benefit of the relief intensity has often been discussed via changes in blood lactate concentration [50]; however, neither blood [51] nor muscle lactate has a direct (nor linear) relationship with performance capacity [50]. It has also been shown that substantially different intermittent training modalities (as assessed by accumulated Bla-1 levels and the HR) may have relatively similar muscle mean peripheral O_2_ responses.

### 4.2. O_2_ Responses in Different Muscles

Information about simultaneous oxygenation in different muscles provides a potential understanding of internal load. Paquette and Bieuzen [30] aimed to understand muscle oxygenation in more than one active muscle and suggested that the maximum O_2_ extraction is independent and a better performance predictor than the VO_2_max in sprint canoeing and kayaking. Thus, our main results on muscle oxygenation during the RT show differences between SmO_2_ in the LD, PM, and VL and between the tHb in the VL, PM, and LD during all workout intervals (Figure 2). This could suggest higher PM recruitment during ergometer paddling than the LD, and especially with the VL, in the applied intermittent training sessions. The Δ tHb was lower in the VL than in the PM and LD across all the training protocols, suggesting a decrease in the leg muscle’s blood volume (Table 4). This is in line with previous studies that showed a higher energy requirement of the fatigued muscle per unit of external work performed than the non-fatigued muscle [52]. The deoxygenation of the LD in the SIT was higher than during the other protocols, which was confirmed by a previous electromyography study on different muscle activation levels during kayak paddling, which showed that the LD muscle is highly active during the draw phase of the kayaking [52]. However, an increase in different muscle activation levels during different training protocols, which will likely produce an increase in O_2_ extraction, may be associated with the technique required to cover the distances of different intensities. The tHb was lower in the VL (12.4 ± 0.1) than in the PM (13.2 ± 0.1) and LD (13.3 ± 0.1), suggesting a decrease in the muscle blood volume in lower body muscles. The drop in O_2_ saturation in the less active muscles is explained by the sympathetic flow induced by exercise, promoting vasoconstriction in this tissue and consequently, a redirection of the blood flow to the more active muscles [53]. This way of explanation about the decreased muscle oxygenation in the non-exercising limb was already used during graded leg cycling exercises, by adopting ultrasound and NIRS methods [54]. At the same time, we did not observe any differences in the tHb between the RT, IT, and SIT in the LD during the exercise bouts and in the VL during the rest bouts, which should be considered in future studies.

Despite some limitations of the NIRS technique and its technology [55], this study was conducted during a real training scenario in the preparatory training period for the world-class kayak competition. Our study was limited to one participant to find out the individual response to single kayak training. Intermittent training is associated with aerobic and anaerobic metabolism; therefore, for practical reasons, it was not possible to invasively determine the accumulation of Bla after each exercise bout by measuring this level at the end of the training. However, previous studies of elite kayakers have shown [56] that the mean lactate threshold occurred at a blood lactate concentration of 2.7 mmol·L^−1^, an HR of 170 beats·min^−1^, and a VO_2_ of 44.2 mL·kg^−1^·min^−1^. The lactate threshold presented corresponded to a percentage of 89.6% of the maximum heart rate and 82.4% of the VO_2_ peak. This shows that the characteristics of our subject are close to these indicators. Therefore, the relationships between oxygen kinetics and anaerobic metabolism should be further examined with experimental training studies.

## 5. Conclusions

The current results suggest that the observations of intermittent exercise performance and significant changes in the peripheral effect of muscle oxygenation in response to training stimuli are the internal predictors of the aerobic metabolism intensity related to work, relief, and recovery intensity. Differences in muscle oxygenation suggest muscle recruitment between the PM, LD, and VL during different exercises; however, this area is still poorly understood requiring further research. To our knowledge, this is the first study that shows the significant contribution of the PM muscle on individual performance in world-class kayakers following different modality intermittent kayak training. In addition to the HR, blood lactate, and VO_2_ measurements, wearable NIRS technology is, therefore, a significant tool for monitoring muscle oxidative metabolism during different training modalities.

## Figures and Tables

**Table 1:** Subject’s physical characteristics.

VO_2_max (L·min^−1^)	5.1
VO_2_max (mL·kg^−1^·min^−1^)	57.5
HR (beat·min^−1^) at CIL	180.0
Power (W) at CIL	300.0
VO_2_ (L·min^−1^) at VT2	3.8
VO_2_ (mL·kg^−1^·min^−1^) at VT2	43.0
HR (beat·min^−1^) at VT2	165.0
Power (W) at VT2	200.0

Note: VO_2_max—maximal oxygen uptake; CIL—critical intensity limit; VT2—2nd ventilatory threshold; HR—heart rate; W—watts.

**Table 2:** Experimental conditions of the protocol depicting the three IT intensity modes.

Duration	15′	6 Bouts	
RT	Warm-Up	S-6′	6′
	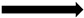	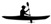	PR
	5′-160 W + 1,30′PR + 3,30′-3X(10″300 W + 1′PR) + 5′PR	I-200 W	
IT	Warm-Up	S-60″ + 60″	6′
	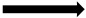	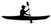	PR
	5′-160 W + 1,30′PR + 3,30′-3X(10″300 W + 1′PR) + 5′PR	I-200 W + 40 W	
	Warm-Up	S-10″ + 30″	6′
SIT	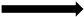	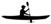	PR
	5′-160 W + 1,30′PR + 3,30′-3X(10″300 W + 1′PR) + 5′PR	I-300 W + 40 W	

Note: RT—repetition training protocol 1; IT—interval training protocol 2; SIT—sprint interval training protocol 3; S—series; PR—passive recovery; W—watts; I—intensity.

**Table 3:** Characterization and inferential analysis of the considered variables according to different intermittent training protocols during exercise bouts.

Bouts	Variables	Intermittent Training Protocol			Difference in Means (Row; ±95% CL)			
		RT	IT	SIT	(a)	(b)	(c)	*p*
		Mean ± SD	Mean ± SD	Mean ± SD				
	SmO_2_ (%) LD	43.4 ± 4.8	46.1 ± 6.8	40.5 ± 4.1	2.7 [−1.1, 6.6]	−2.9 [−5, −0.8]	−5.6 [−10.4, −0.9]	<0.05
	tHb (AU) LD	13.2 ± 0	13.3 ± 0.1	13.2 ± 0.1	0.1 [0, 0.2]	0.1 [0, 0.1]	0 [−0.1, 0]	<0.05
	SmO_2_ (%) PM	30.7 ± 8.9	44.2 ± 10.0	37.0 ± 5.0	13.5 [6.2, 20.9]	6.3 [3.4, 9.2]	−7.2 [−14.1, −0.3]	<0.01
1	tHb (AU) PM	13.2 ± 0	13.2 ± 0.1	13.1 ± 0	0 [−0.1, 0]	−0.1 [−0.1, −0.1]	0 [−0.1, 0]	<0.01
	SmO_2_ (%) VL	64.8 ± 5.7	77.3 ± 4.0	70.4 ± 3.6	12.4 [7.3, 17.6]	5.6 [3.8, 7.4]	−6.8 [−10.5, −3.1]	<0.001
	tHb (AU) VL	12.4 ± 0.1	12.4 ± 0.1	12.3 ± 0	0.1 [0, 0.1]	−0.1 [−0.1, −0.1]	−0.1 [−0.2, −0.1]	<0.01
	HR (Bpm)	144.7 ± 11.4	122.2 ± 13.3	127.7 ± 3.9	−22.5 [−31.5, −13.5]	−17.0 [−22.8, −11.2]	5.5 [−3, 13.9]	<0.001
	SmO_2_ (%) LD	47.3 ± 3.8	47.2 ± 7.9	42.5 ± 3.8	−0.1 [−4.4, 4.3]	−4.8 [−6.8, −2.7]	−4.7 [−9.4, 0]	<0.05
	tHb (AU) LD	13.2 ± 0.1	13.3 ± 0.2	13.3 ± 0.1	0.1 [−0.1, 0.2]	0.1 [0, 0.2]	0.1 [0, 0.2]	>0.05
	SmO_2_ (%) PM	32.5 ± 8.6	42.6 ± 10.6	38.2 ± 5.3	10.1 [3.3, 16.9]	5.7 [2.4, 8.9]	−4.4 [−11.5, 2.7]	<0.05
2	tHb (AU) PM	13.2 ± 0	13.2 ± 0	13.1 ± 0	−0.1 [−0.1, 0]	−0.1 [−0.1, −0.1]	0 [−0.1, 0]	<0.001
	SmO_2_ (%) VL	63.6 ± 5.7	78.4 ± 4.1	70.6 ± 2.8	14.8 [9.5, 20.1]	7 [4.7, 9.2]	−7.8 [−11.3, −4.4]	<0.001
	tHb (AU) VL	12.4 ± 0.1	12.4 ± 0.1	12.3 ± 0	0 [0, 0.1]	−0.1 [−0.1, −0.1]	−0.1 [−0.2, −0.1]	<0.01
	HR (Bpm)	151.3 ± 11.4	120.4 ± 12.8	127.0 ± 7.4	−30.9 [−40.8, −21.0]	−24.3 [−27.1, −21.5]	6.6 [−2.5, 15.8]	<0.001
	SmO_2_ (%) LD	48.8 ± 3.3	49.4 ± 6.9	43.9 ± 47	0.7 [−2.3, 3.7]	−4.8 [−6.3, −3.3]	−5.5 [−8.8, −2.2]	<0.001
	tHb (AU) LD	13.3 ± 0.1	13.4 ± 0.2	13.4 ± 0.1	0.1 [0, 0.3]	0.1 [0, 0.1]	−0.1 [−0.2, 0.1]	>0,.05
	SmO_2_ (%) PM	34.0 ± 8.3	45.2 ± 9.1	41.5 ± 4.5	11.2 [5.2, 17.2]	7.5 [4.4, 10.5]	−3.7 [−10.0, 2.5]	<0.01
3	tHb (AU) PM	13.2 ± 0	13.2 ± 0	13.2 ± 0	0 [−0.1, 0]	−0.1 [−0.1, 0]	0 [0, 0]	<0.01
	SmO_2_ (%) VL	62.4 ± 6.3	78.9 ± 4.0	71.2 ± 3.5	16.6 [10.8, 22.3]	8.8 [6.7, 11.0]	−7.7 [−11.7, −3.8]	<0.001
	tHb (AU) VL	12.4 ± 0.1	12.4 ± 0.1	12.3 ± 0	0 [0, 0.1]	−0.1 [−0.1, −0.1]	−0.1 [−0.2, −0.1]	<0.01
	HR (Bpm)	154.6 ± 11.3	120.9 ± 14.2	125.8 ± 5.0	−33.7 [−44.2, −23.2]	−28.8 [−33.4, −24.3]	4.9 [−5.1, 14.9]	<0.001
	SmO_2_ (%) LD	49.1 ± 4.1	50.8 ± 5.7	43.8 ± 3.8	1.7 [−0.8, 4.2]	−5.3 [−7.1, −3.5]	−7.0 [−9.9, −4.1]	<0.001
	tHb (AU) LD	13.4 ± 0.1	13.5 ± 0.2	13.3 ± 0.1	0.1 [−0.1, 0.2]	0 [−0.1, 0.1]	−0.1 [−0.2, 0]	>0.05
	SmO_2_ (%) PM	29.6 ± 9.4	48.6 ± 9.7	36.9 ± 6.2	19.0 [12.2, 25.7]	7.3 [4.2, 10.4]	−11.7 [−18.6, −4.7]	<0.001
4	tHb (AU) PM	13.2 ± 0	13.3 ± 0	13.1 ± 0	0.1 [0, 0.1]	−0.1 [−0.1, 0]	−0,.1 [−0.1, −0.1]	<0.001
	SmO_2_ (%) VL	61.0 ± 6.2	77.4 ± 4.5	72.5 ± 4.1	16.4 [10.7, 22.0]	11.5 [9.7, 13.3]	−4.9 [−9.3, −0.5]	<0.001
	tHb (AU) VL	12.4 ± 0	12.4 ± 0.1	12.3 ± 0.1	0 [0, 0.1]	−0.1 [−0.1, −0.1]	−0.1 [−0.2, 0]	<0.01
	HR (Bpm)	158.0 ± 10.9	118.8 ± 14.0	126.2 ± 9.3	−39.2 [−48.7, −29.7]	−31.7 [−34.5, −28.9]	7.5 [−1.8, 16.7]	<0.001
	SmO_2_ (%) LD	48.0 ± 3.4	50.9 ± 6.1	39.9 ± 4.6	2.9 [−0.2, 5.9]	−8.2 [−10.1, −6.2]	−11.0 [−15.1, −7.0]	<0.001
	tHb (AU) LD	13.3 ± 0.1	13.5 ± 0.2	13.3 ± 0.1	0.1 [0, 0.3]	0 [−0.1, 0]	−0.2 [−0.3, 0]	<0.05
	SmO_2_ (%) PM	26.3 ± 8.1	47.6 ± 9.6	37.6 ± 6.1	21.3 [15.5, 27.1]	11.2 [8.5, 13.9]	−10.1 [−17.2, −2.9]	<0.001
5	tHb (AU) PM	13.2 ± 0	13.2 ± 0	13.1 ± 0	0.1 [0, 0.1]	−0.1 [−0.1, 0]	−0.1 [−0.1, −0.1]	<0.001
	SmO_2_ (%) VL	60.4 ± 6.3	78.4 ± 3.7	73.3 ± 3.9	18.0 [12.6, 23.4]	13.0 [11.1, 14.8]	−5.0 [−9.2, −0.9]	<0.001
	tHb (AU) VL	12.4 ± 0	12.4 ± 0.1	12.3 ± 0.1	0 [0, 0.1]	−0.1 [−0.1, −0.1]	−0.1 [−0.2, −0.1]	<0.001
	HR (Bpm)	159.8 ± 11.2	119.5 ± 14.7	127.9 ± 6.3	−40.3 [−50.8, −29.7]	−31.9 [−35.6, −28.1]	8.4 [−2.1, 19.0]	<0.001
	SmO_2_ (%) LD	47.9 ± 3.6	50.0 ± 5.3	40.9 ± 3.8	2.2 [−0.4, 4.7]	−7.0 [−8.6, −5.4]	−9.2 [−11.7, −6.6]	<0.001
	tHb (AU) LD	13.3 ± 0.1	13.3 ± 0.2	13.3 ± 0.1	0.1 [−0.1, 0.2]	0 [−0.1, 0]	−0.1 [−0.2, 0.1]	>0.05
	SmO_2_ (%) PM	33.2 ± 8.0	46.9 ± 9.6	36.1 ± 5.6	13.7 [8.0, 19.4]	2.9 [0.6, 5.1]	−10.8 [−17.1, −4.6]	<0.001
6	tHb (AU) PM	13.2 ± 0	13.2 ± 0	13.1 ± 0	0 [−0.1, 0]	−0.1 [−0.1, −0.1]	−0.1 [−0.1, −0.1]	<0.001
	SmO_2_ (%) VL	61.2 ± 6.6	80.4 ± 2.3	73.6 ± 3.7	19.2 [14.4, 24.1]	12.5 [10.3, 14.7]	−6.8 [−9.7, −3.8]	<0.001
	tHb (AU) VL	12.4 ± 0.1	12.5 ± 0.1	12.3 ± 0.1	0.1 [0.1, 0.2]	−0.1 [−0.1, −0.1]	−0.2 [−0.3, −0.2]	<0.001
	HR (Bpm)	161.8 ± 10.7	119.4 ± 14.4	129.4 ± 6.3	−42.4 [−52.5, −32.3]	−32.4 [−36.0, −28.8]	10.0 [−0.1, 20.0]	<0.001

Note: SmO_2_, muscle O_2_ saturation; tHb, total hemoglobin; HR, heart rate; LD, latissimus dorsi; PM, pectoralis major; VL, vastus lateralis; CL = confidence limits; RT, repetition training; IT, interval training; SIT, sprint interval training. Comparisons among intermittent training protocols during exercise bouts identified as (a) RT vs. IT, (b) RT vs. SIT, and (c) IT vs. SIT.

**Table 4:** Characterization and inferential analysis of the considered variables according to different intermittent training protocols during rest bouts.

Bouts	Variables	Intermittent Training Protocol			Difference in Means (Row; ±95% CL)			
		RT	IT	SIT	(a)	(b)	(c)	*p*
		Mean ± SD	Mean ± SD	Mean ± SD				
	SmO_2_ (%) LD	65.7 ± 7.4	70.2 ± 6.9	71.2 ± 6.7	4.5 [1.0, 8.0]	5.5 [2.5, 8.5]	1.0 [−0.7, 2.7]	<0.01
	tHb (AU) LD	13.1 ± 0	13.2 ± 0.1	13.2 ± 0	0.1 [0, 0.1]	0.1 [0.1, 0.2]	0 [0, 0.1]	<0.001
	SmO_2_ (%) PM	64.7 ± 12.1	72.7 ± 4.7	72.0 ± 9.7	8.0 [2.7, 13.3]	7.4 [2.6, 12.1]	−0.7 [−4.6, 3.3]	<0.01
1	tHb (AU) PM	13.0 ± 0	13.1 ± 0	13.0 ± 0	0.1 [0.1, 0.2]	0.1 [0, 0.1]	−0.1 [−0.1, 0]	<0.001
	SmO_2_ (%) VL	74.9 ± 3.4	77.4 ± 3.5	78.6 ± 2.8	2.6 [−1.0, 6.1]	3.8 [2.5, 5.1]	1.2 [−2.0, 4.4]	<0.05
	tHb (AU) VL	12.4 ± 0.1	12.4 ± 0	12.4 ± 0	0 [0, 0.1]	0.1 [0, 0.1]	0 [0, 0.1]	>0.05
	HR (Bpm)	95.6 ± 16.7	77.7 ± 7.4	77.6 ± 8.4	−17.9 [−25.8, −10.0]	−18.0 [−25.7, −10.4]	−0.2 [−6.5, 6.1]	<0.001
	SmO_2_ (%) LD	64.5 ± 7.0	70.3 ± 6.8	69.0 ± 7.8	5.8 [3.0, 8.6]	4.6 [2.0, 7.2]	−1.2 [−2.6, 0.2]	<0.001
	tHb (AU) LD	13.1 ± 0	13.2 ± 0.1	13.2 ± 0.1	0.1 [0.1, 0.2]	0.1 [0.1, 0.2]	0 [0, 0]	<0.001
	SmO_2_ (%) PM	65.9 ± 11.5	72.0 ± 6.5	71.4 ± 9.2	6.1 [1.5, 10.6]	5.5 [2.0, 9.0]	−0.6 [−2.7, 1.6]	<0.01
2	tHb (AU) PM	13.1 ± 0	13.0 ± 0	13.1 ± 0	0 [−0.1, 0]	0 [0, 0]	0 [0, 0.1]	<0.05
	SmO_2_ (%) VL	74.8 ± 4.3	79.5 ± 3.6	77.8 ± 2.6	4.7 [0.7, 8.7]	3.0 [1.0, 5.0]	−1.7 [−5.2, 1.7]	<0.05
	tHb (AU) VL	12.4 ± 0.1	12.4 ± 0.1	12.4 ± 0	0 [−0.1, 0.1]	0 [−0.1, 0]	0 [−0.1, 0]	>0.05
	HR (Bpm)	101.6 ± 18.1	73.1 ± 6.2	77.5 ± 10.8	−28.5 [−37.0, −20.0]	−24.1 [−30.3, −17.9]	4.4 [0.6, 8.1]	<0.001
	SmO_2_ (%) LD	63.6 ± 7.0	68.4 ± 6.3	67.3 ± 7.3	4.8 [1.6, 8.0]	3.7 [1.1, 6.3]	−1.1 [−2.8, 0.5]	<0.01
	tHb (AU) LD	13.1 ± 0.1	13.3 ± 0.1	13.2 ± 0	0.1 [0.1, 0.2]	0.1 [0, 0.1]	0 [−0.1, 0]	<0.001
	SmO_2_ (%) PM	64.7 ± 11.7	73.5 ± 4.6	70.8 ± 8.1	8.8 [4.0, 13.7]	6.1 [2.0, 10.1]	−2.8 [−5.3, −0.2]	<0.001
3	tHb (AU) PM	13.0 ± 0.1	13.1 ± 0.1	13.1 ± 0	0.1 [0, 0.2]	0 [0, 0]	−0.1 [−0.1, 0]	<0.01
	SmO_2_ (%) VL	73.4 ± 4.4	77.0 ± 4.3	79.0 ± 2.4	3.5 [−0.6, 7.6]	5.6 [4.1, 7.1]	2.1 [−1.5, 5.6]	<0.05
	tHb (AU) VL	12.4 ± 0	12.4 ± 0.1	12.4 ± 0	0 [0, 0.1]	0 [0, 0.1]	0 [−0.1, 0.1]	>0.05
	HR (Bpm)	107.1 ± 17.4	77.2 ± 7.8	79,9 ± 10.7	−29.9 [−38.9, −20.9]	−27.2 [−34.7, −19.7]	2.7 [−1.5, 6.9]	<0001
	SmO_2_ (%) LD	62.5 ± 6.3	67.5 ± 6.9	67.4 ± 7.0	4.9 [1.8, 8.0]	4.8 [2.5, 7.2]	−0.1 [−1.6, 1.4]	<0.001
	tHb (AU) LD	13.1 ± 0.1	13.2 ± 0.1	13.2 ± 0	0.1 [0, 0.2]	0.1 [0.1, 0.2]	0 [−0.1, 0]	<0.001
	SmO_2_ (%) PM	62.5 ± 12.1	75.3 ± 4.9	69.4 ± 9.7	12.8 [7.8, 17.8]	6.8 [3.6, 10.0]	−6.0 [−9.2, −2.7]	<0.001
4	tHb (AU) PM	13.0 ± 0.1	13.2 ± 0	13.0 ± 0	0.2 [0.1, 0.2]	0 [0, 0]	−0.2 [−0.2, −0.2]	<0.001
	SmO_2_ (%) VL	74.9 ± 5.2	78.5 ± 4.4	80.0 ± 2.0	3.5 [−1.5, 8.5]	5.1 [2.9, 7.3]	1.5 [−2.0, 5.0]	<0.05
	tHb (AU) VL	12.4 ± 0	12.4 ± 0.1	12.4 ± 0	0 [0, 0.1]	0 [0, 0]	0 [−0.1, 0]	>0.05
	HR (Bpm)	104.4 ± 18.6	74.0 ± 7.5	84.1 ± 8.3	−30.4 [−39.8, −21.1]	−20.3 [−30.6, −10.0]	10.1 [3.4, 16.9]	<0.001
	SmO_2_ (%) LD	60.5 ± 8.2	68.0 ± 6.7	67.3 ± 8.1	7.4 [4.3, 10.5]	6.8 [4.2, 9.3]	−0.7 [−1.9, 0.6]	<0.001
	tHb (AU) LD	13.1 ± 0	13.2 ± 0	13.2 ± 0	0.2 [0.1, 0.2]	0.1 [0.1, 0.2]	0 [0, 0]	<0.001
	SmO_2_ (%) PM	63.5 ± 13.4	74.7 ± 6.5	71.4 ± 10.6	11.2 [6.2, 16.2]	7.9 [4.0, 11.8]	−3.3 [−6.2, −0.4]	<0.001
5	tHb (AU) PM	13.1 ± 0	13.1 ± 0	13.1 ± 0	0.1 [0, 0.1]	0 [0, 0]	−0.1 [−0.1, 0]	<0.001
	SmO_2_ (%) VL	76.5 ± 5.9	81.0 ± 3.6	80.8 ± 2.3	4.5 [−0.7, 9.6]	4.4 [1.8, 6.9]	−0.1 [−3.3, 3.0]	>0.05
	tHb (AU) VL	12.4 ± 0	12.5 ± 0.1	12.4 ± 0	0.1 [0.1, 0.2]	0 [0, 0.1]	−0.1 [−0.1, 0]	<0.001
	HR (Bpm)	107.7 ± 19.4	72.5 ± 8.3	77.3 ± 7.7	−35.2 [−43.2, −27.1]	−30.4 [−38.8, −22.0]	4.8 [1.1, 8.4]	<0.001
	SmO_2_ (%) LD	58.3 ± 7.2	65.4 ± 7.6	61.0 ± 4.0	7.2 [2.5, 11.9]	2.7 [−0.5, 5.9]	−4.5 [−7.7, −1.2]	<0.01
	tHb (AU) LD	13.1 ± 0.1	13.2 ± 0.1	13.2 ± 0	0.1 [0, 0.1]	0.1 [0.1, 0.1]	0 [0, 0.1]	<0.001
	SmO_2_ (%) PM	54.3 ± 7.9	70.8 ± 6.1	63.5 ± 8.2	16.5 [13.4, 19.7]	9.2 [6.2, 12.3]	−7.3 [−9.7, −4.9]	<0.001
6	tHb (AU) PM	13.0 ± 0.1	13.1 ± 0	13.0 ± 0	0.1 [0, 0.2]	0 [−0.1, 0]	−0.1 [−0.1, −0.1]	<0.01
	SmO_2_ (%) VL	71.1 ± 5.5	80.4 ± 3.5	76.3 ± 2.4	9.3 [5.7, 13.0]	5.2 [1.4, 8.9]	−4.2 [−7.2, −1.2]	<0.01
	tHb (AU) VL	12.5 ± 0.1	12.5 ± 0	12.4 ± 0.1	0 [−0.1, 0.1]	−0.1 [−0.1, 0]	−0.1 [−0.1, 0]	>0.05
	HR (Bpm)	116.4 ± 16.6	75.4 ± 6.7	85.3 ± 10.7	−41.1 [−49.0, −33.2]	−31.2 [−38.5, −23.8]	9.9 [3.5, 16.3]	<0.001

Note: LD, latissimus dorsi; PM, pectoralis major; VL, vastus lateralis; CL = confidence limits; RT, repetition training; IT, interval training; SIT, sprint interval training. Comparisons among intermittent training protocols during exercise bouts identified as (a) RT vs. IT, (b) RT vs. SIT, and (c) IT vs. SIT.

**Table 5:** Changes in Δ SmO_2_ (%) and Δ tHb (AU) following different training protocols for each bout.

Bouts	Variables	Intermittent Training Protocol		
		RT	IT	SIT
1	Δ SmO_2_ (%) LD	−44.36	−38.48	−49.53
	Δ tHb (AU) LD	0.18	0.46	0.56
	Δ SmO_2_ (%) PM	−58.74	−36.71	−45.25
	Δ tHb (AU) PM	0.27	0.37	0.23
	Δ SmO_2_ (%) VL	−13.30	−2.24	−4.13
	Δ tHb (AU) VL	0.21	0.29	0.01
2	Δ SmO_2_ (%) LD	−47.65	−38.48	−50.80
	Δ tHb (AU) LD	0.46	0.60	1.10
	Δ SmO_2_ (%) PM	−54.04	−35.46	−42.93
	Δ tHb (AU) PM	0.29	0.31	0.25
	Δ SmO_2_ (%) VL	−15.96	−2.24	−6.51
	Δ tHb (AU) VL	0.21	0.33	0.06
3	Δ SmO_2_ (%) LD	−48.38	−33.87	−50.17
	Δ tHb (AU) LD	0.77	0.85	0.76
	Δ SmO_2_ (%) PM	−48.39	−31.71	−40.25
	Δ tHb (AU) PM	0.32	0.28	0.26
	Δ SmO_2_ (%) VL	−17.21	−2.29	−9.00
	Δ tHb (AU) VL	0.21	0.37	0.09
4	Δ SmO_2_ (%) LD	−43.87	−32.99	−44.91
	Δ tHb (AU) LD	1.02	0.78	0.59
	Δ SmO_2_ (%) PM	−63.33	−32.56	−45.82
	Δ tHb (AU) PM	0.28	0.41	0.24
	Δ SmO_2_ (%) VL	−18.46	−3.41	−6.51
	Δ tHb (AU) VL	0.19	0.20	0.14
5	Δ SmO_2_ (%) LD	−44.04	−38.18	−52.17
	Δ tHb (AU) LD	0.42	0.85	0.71
	Δ SmO_2_ (%) PM	−60.83	−30.46	−43.46
	Δ tHb (AU) PM	0.27	0.34	0.21
	Δ SmO_2_ (%) VL	−20.96	−2.21	−2.94
	Δ tHb (AU) VL	0.19	0.22	0.11
6	Δ SmO_2_ (%) LD	−45.20	−36.53	−51.66
	Δ tHb (AU) LD	0.44	0.69	0.83
	Δ SmO_2_ (%) PM	−56.86	−30.46	−48.31
	Δ tHb (AU) PM	0.33	0.36	0.18
	Δ SmO_2_ (%) VL	−19.47	−2.19	−2.98
	Δ tHb (AU) VL	0.22	0.42	0.14

Note: LD—latissimus dorsi; PM—pectoralis major; VL—vastus lateralis; RT—repetition training; IT—interval training; SIT—sprint interval training.

## Data Availability

The datasets used and/or analyzed during the current study are publicly available from the corresponding author upon reasonable request. Interested researchers may contact the board from the Vytautas Magnus University to request access to the data (VMU, info@vdu.lt).
